# Tum1 is involved in the metabolism of sterol esters in *Saccharomyces cerevisiae*

**DOI:** 10.1186/s12866-017-1088-1

**Published:** 2017-08-22

**Authors:** Katja Uršič, Mojca Ogrizović, Dušan Kordiš, Klaus Natter, Uroš Petrovič

**Affiliations:** 1 Jožef Stefan Institute, Department of Molecular and Biomedical Sciences, Jamova cesta 39, 1000 Ljubljana, Slovenia; 2 University of Graz, Institute of Biomolecular Sciences, Humboldtstraße 50/EG, 8010 Graz, Austria; 3 Biotechnical Faculty, Department of Biology, University of Ljubljana, Večna pot 111, 1000 Ljubljana, Slovenia; 4 Present address: Institute of Oncology Ljubljana, Zaloška cesta 2, 1000 Ljubljana, Slovenia

**Keywords:** Lipid metabolism, tRNA modification, Yeast *Saccharomyces cerevisiae*

## Abstract

**Background:**

The only hitherto known biological role of yeast *Saccharomyces cerevisiae* Tum1 protein is in the tRNA thiolation pathway. The mammalian homologue of the yeast *TUM1* gene, the thiosulfate sulfurtransferase (a.k.a. rhodanese) *Tst*, has been proposed as an obesity-resistance and antidiabetic gene. To assess the role of Tum1 in cell metabolism and the putative functional connection between lipid metabolism and tRNA modification, we analysed evolutionary conservation of the rhodanese protein superfamily, investigated the role of Tum1 in lipid metabolism, and examined the phenotype of yeast strains expressing the mouse homologue of Tum1, TST.

**Results:**

We analysed evolutionary relationships in the rhodanese superfamily and established that its members are widespread in bacteria, archaea and in all major eukaryotic groups. We found that the amount of sterol esters was significantly higher in the deletion strain *tum1*Δ than in the wild-type strain. Expression of the mouse TST protein in the deletion strain did not rescue this phenotype. Moreover, although Tum1 deficiency in the thiolation pathway was complemented by re-introducing *TUM1*, it was not complemented by the introduction of the mouse homologue *Tst*. We further showed that the tRNA thiolation pathway is not involved in the regulation of sterol ester content in *S. cerevisiae*, as overexpression of the tE^UUC^, tK^UUU^ and tQ^UUG^ tRNAs did not rescue the lipid phenotype in the *tum1*Δ deletion strain, and, additionally, deletion of the key gene for the tRNA thiolation pathway, *UBA4*, did not affect sterol ester content.

**Conclusions:**

The rhodanese superfamily of proteins is widespread in all organisms, and yeast *TUM1* is a bona fide orthologue of mammalian *Tst* thiosulfate sulfurtransferase gene. However, the mouse TST protein cannot functionally replace yeast Tum1 protein, neither in its lipid metabolism-related function, nor in the tRNA thiolation pathway. We show here that Tum1 protein is involved in lipid metabolism by decreasing the sterol ester content in yeast cells, and that this function of Tum1 is not exerted through the tRNA thiolation pathway, but through another, currently unknown pathway.

## Background

Lipids are important dynamic molecules that play vital roles, which extend beyond their structural roles in cellular membranes [1–4]. Lipid metabolism has lately gained much attention in particular due to its role in health and disease, especially in the emerging pandemics of obesity and type 2 diabetes [5, 6]. Recently, mouse thiosulfate sulfurtransferase (TST) protein was proposed to have obesity-resistance and anti-diabetes effects in mammals [7]. The mechanism of action causing the decreased accumulation of storage lipids was proposed to be increased expression and activity of the protein, resulting in augmented mitochondrial function and increased degradation of reactive oxygen species and sulfide.

*TUM1* (*YOR251c*) is a unique yeast *Saccharomyces cerevisiae* ortholog of the human *TST* gene [8]. Tum1 and TST/MPST (mercaptopyruvate sulfurtransferase) proteins belong to the rhodanese/cell cycle control phosphatase protein superfamily. The rhodanese superfamily contains the fold with the same name and the proteins from this superfamily span a wide range of molecular functions. In this study we examined the molecular role of the yeast Tum1 protein which has been previously described only as a protein with a non-essential function in the tRNA wobble uridine modification pathway [9, 10]. This pathway consists also of Uba4, Ncs2, Ncs6 and Nfs1 proteins and is required for the thiolation of three tRNA molecules, specific for glutamine (tQ^UUG^), lysine (tK^UUU^), and glutamic acid (tE^UUC^) tRNAs, which contain 2-thiouridine derivate as the wobble nucleoside [11]. To date, 156 genes have been annotated to lipid metabolism in *S. cerevisiae* [12] and additional proteins are still being described as involved in this complex process [4]. However, no functional connection between tRNA modification and lipid metabolism has been established yet, opening a question of structural and functional conservation of the rhodanese superfamily members in general, and Tum1 and TST in particular. We therefore analysed evolutionary conservation of the rhodanese protein superfamily, investigated the role of Tum1 in lipid metabolism, and examined the putative functional connection between lipid metabolism and tRNA modification processes.

## Methods

### Sequence data mining

All database searches were performed online. The databases analysed were the prokaryotic and eukaryotic protein, transcriptome and genome databases at the National Center for Biotechnology Information [13]. To detect all available representatives of the rhodanese superfamily, database searches were performed iteratively. Comparisons were performed using the TBLASTN program [14], with the E-value cutoff set to 10^−5^ and default settings for other parameters. Diverse prokaryotic and eukaryotic representatives of the rhodanese superfamily [15, 16] have been used as queries. The Translate program from Expasy [17] was used to translate DNA sequences.

### Phylogenetic analysis

Eukaryotic and prokaryotic representatives of the rhodanese superfamily [15, 16] were included in the analyses. Diverse opisthokont representatives of the rhodanese superfamily were also included in the separate analysis. The rhodanese domain in the newly discovered representatives of the rhodanese superfamily was identified by using the SMART [18], InterPro [19] and Pfam [20] domain databases. The protein sequences were aligned using Clustal Omega [21]. We used IQ Tree web server [22] to select the best maximum likelihood model of sequence evolution for the data from the rhodanese superfamily. The best fit model of amino acid substitution for our data set was determined as LG + G4 according to BIC (Bayesian information criterion). Phylogenetic trees were reconstructed using the maximum likelihood method [23] and the neighbor-joining method [24]. The reliability of the resulting topologies was tested by the bootstrap method. Two archaeal rhodanese superfamily representatives were used as the outgroup in the global rhodanese phylogeny, while the *Arabidopsis* TST1 repeat domain of rhodanese protein was used to root the *Opisthokonta* rhodanese tree. Phylogenetic analyses were performed with IQ Tree web server [22].

### Synteny analysis of the rhodanese superfamily in vertebrates

For synteny analyses, the chromosomal locations, lengths, and the directionality of the neighbouring genes upstream and downstream of *Tst* and *Mpst* genes were extracted from Ensembl [25] and Entrez [26] genome databases. Additional synteny comparisons were conducted using Genomicus [27].

### Strains and media

The *Saccharomyces cerevisiae* wild-type (WT) strain BY4741 (*MATa his3∆1 leu2∆0 met15∆ ura3∆0*) and the isogenic *uba4*Δ deletion strain were obtained from the EUROSCARF strain collection. The *tum1*Δ deletion strain was constructed by replacing the *TUM1* open reading frame (ORF) with the *KanMX* resistance cassette. A strain with re-inserted *TUM1* was used as the control strain. The ORF of the *Tst* gene was PCR-amplified from the appropriate mouse (*Mus musculus*) cDNA clone and introduced into the *TUM1* locus, under the control of the endogenous yeast promoter.

Strains were grown in rich medium YPD (2% peptone, 1% yeast extract, 2% glucose) or in synthetic complete medium lacking uracil (SC-Ura). YPD plates containing 0.5 mg/ml geneticin (G418, Formedium) or 0.1 mg/ml nourseothricin (clonNAT, Werner) were used to select for geneticin or nourseothricin resistance.

### Plasmid construction

DNA manipulations, plasmid preparations and bacterial transformations were performed according to standard methods. For expression of *TUM1* or *Tst* under control of the *ADH1* promoter, the respective ORFs were inserted into the pUG35 plasmid backbone [28] immediately after the *ADH1* promoter. Plasmids were named according to inserts: [empty] for backbone only, [*TUM1*] for the plasmid expressing yeast *TUM1* and [*Tst*] for the plasmid expressing mouse *Tst*.

Plasmids pABY1653 (tE^UUC^-tK^UUU^-tQ^UUG^) and pABY525 (control), described in [29], were kindly provided by Prof. Anders Byström, Umeå University, Sweden.

### Isolation and purification of tRNA

Yeast strains were grown in 100 ml of YPD or SC-Ura medium and harvested during exponential phase of growth (OD_600_ = 0.5–0.6). Total RNA was isolated using hot acid phenol chloroform extraction. We separated tRNA molecules from other types of RNA molecules as described [30]. The isolated tRNA molecules were further purified by denaturing polyacrylamide gel electrophoresis containing 10 μg/ml N-acryloylaminophenylmercuric chloride (APM). The presence of thiouridine in tRNAs was determined by the decrease of electrophoretic mobility on polyacrylamide gel.

### Isolation of proteins, SDS-PAGE and western blot

Yeast strains were grown in 20 ml of SC-Ura medium and harvested during exponential growth phase (OD_600_ = 0.5–0.6). Proteins were isolated as described [31]. Mouse liver tissue whole-cell homogenates were made with RIPA buffer (1% NP-40, 0.1% SDS, 0.5% Na-Deoxycholate in PBS) with added protease inhibitors and used directly for western blot analysis. Yeast and mouse protein mixtures were separated in duplicates on a 12.5% SDS gel with a 4% stacking gel at 30 mA and 300 V. The proteins were transferred to a nitrocellulose membrane, which was afterwards blocked with 5% low fat milk in Tris buffered saline TBS, 0.05% Tween-20 (TBST) for 1 h, and then incubated overnight at 4 °C with the anti-TST primary antibody (EPR1164G(B), RabMAb, 1:1000). After washing with TBST, the membrane was incubated with secondary antibodies in 5% blocking buffer (Goat Anti-rabbit pod, 111–035-003, Jackson Immunoresearch, 1:10,000), and scanned.

### Lipid analysis

Approximately 2 × 10^9^ cells were harvested from exponentially growing cultures (SC medium) and subjected to glass bead disruption in chloroform:methanol 2:1 in a test tube shaker (Heidolph Multi Reax). Lipids were quantitatively extracted with chloroform:methanol 2:1 and analysed by thin layer chromatography (TLC) as described in [32]. For dry weight determination, the cultures were filtrated through 0.45 μm cellulose nitrate filters (Sartorius Stedim) and dried overnight at 95 °C.

### Statistical analysis

Data were tested for normality of distribution by Sharpiro-Wilk test. All pair-wise multiple comparisons were tested using the Holm-Sidak method after one-way analysis of variance. A *p*-value of less than 0.05 was considered to be statistically significant. Data are expressed as average ± standard deviation. SigmaPlot Software (Systat Software Inc) and Excel (Microsoft Office) were used for graphical representation and statistical analysis.

## Results

### Evolutionary relationships in the rhodanese superfamily

**Fig. 1:**
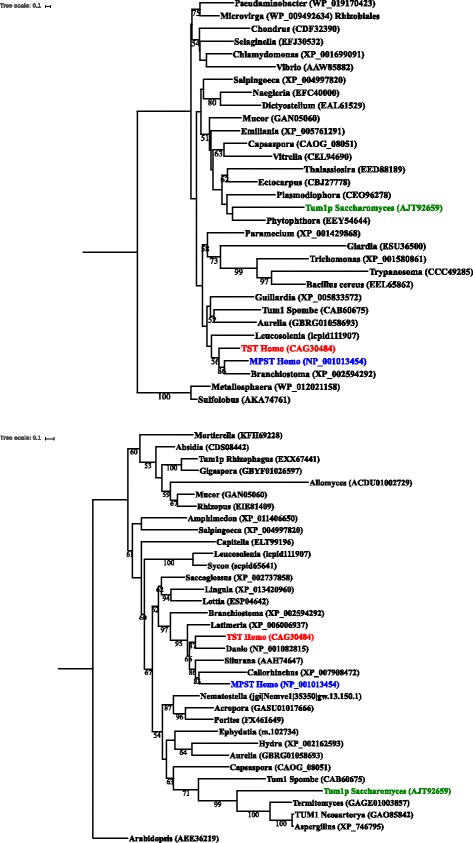
Phylogeny of the rhodanese superfamily. **a** *Global rhodanese phylogeny.* Two archaeal rhodanese representatives were used to root the tree. **b** *Rhodanese superfamily in Opisthokonta.* The TST1 repeat domain of the rhodanese protein from *Arabidopsis* (AEE36219) was used to root this tree. In both cases, the rooted maximum likelihood tree was inferred by IQ Tree [22] under a LG + G4 model from the TST1 repeat domain, and reliability for the internal branches was assessed using the 1000 bootstrap replications. Nodes with confidence values greater than 50% are indicated. All sequences were obtained from the GenBank; genus names and accession numbers are included

To determine evolutionary relationships in the rhodanese superfamily, of which *TUM1* and *Tst* are members, the data available in sequence databases were analysed to resolve the origin and evolutionary history of the superfamily members. The phyletic distribution pattern of the rhodanese superfamily was first analysed. According to global phylogenetic analysis, based on our collection of the representatives, the rhodanese superfamily is widespread in bacteria, archaea and in all major eukaryotic groups (Fig. 1a). Global phylogenetic analysis failed to obtain any evidence for orthologous groups within the rhodanese superfamily (Fig. 1a) and rhodanese representatives from major eukaryotic supergroups mostly did not group in the supergroup manner. The prokaryotic sequences were scattered among the eukaryotic representatives and did not form a monophyletic clade. Moreover, the levels of sequence identity between some prokaryotic and eukaryotic rhodanese representatives reached up to 50% at the amino acid level. The neighbor-joining method with uncorrected distances was found to produce a similar resolution in the rhodanese superfamily as the more complex maximum likelihood method (data not shown).

**Fig. 2:**
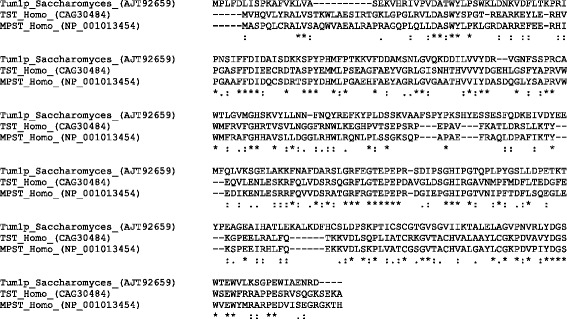
Alignment of yeast Tum1 and human TST and MPST proteins. Alignment was constructed with the program Clustal Omega [21]. Each deletion is indicated by a *dash*. The *asterisks* represent the amino acids conserved between all sequences. *Dots* represent conservative amino acid replacements

In the metazoan and fungi (*Opisthokonta*) tree the representatives of the rhodanese superfamily are relatively well separated (Fig. 1b). Human TST and MPST show 59% amino acid identity, while the human TST compared to yeast Tum1 shows 32% amino acid identity, and human MPST compared to yeast Tum1 shows 37% amino acid identity (Fig. 2).

### Conserved synteny of the rhodanese superfamily in vertebrates

**Fig. 3:**
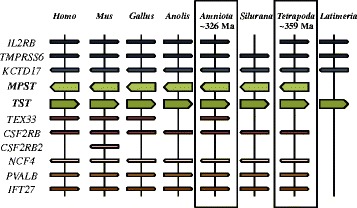
Conserved synteny of the rhodanese superfamily in vertebrates*.* Chromosomal regions carrying *Tst* and *Mpst* genes in the species considered in this analysis were compared, and neighbouring genes with conserved synteny were identified. *Horizontal lines* denote orthologous relationships. Each gene is represented as a *horizontal line* on the chromosome. Neighbouring genes that are in synteny are shown with a schematic indication of their orientation and distance (not to scale). Ancestral states of the *Tst* and *Mpst* chromosomal positions in *Amniota* and *Tetrapoda* were reconstructed from comparisons of syntenic positions between multiple vertebrate lineages

Ancestral states of the *Tst* and *Mpst* chromosomal positions were reconstructed from comparisons of syntenic positions between the diverse vertebrate lineages *Homo, Mus, Gallus, Anolis, Amniota, Silurana* and *Latimeria*. Specifically, the identity and relative genomic position of the genes that map to genomic regions immediately adjacent to either side (5′ vs. 3′) of all vertebrate rhodanese superfamily genes were recorded using the Ensembl and Entrez genome databases as well as the Genomicus database. Analysis of conserved synteny demonstrated that the gene duplication of rhodanese gene superfamily occurred in the ancestor of land vertebrates (~ 359 Mya), producing *Tst* and *Mpst* genes. *Tst* and *Mpst* are duplicated genes, located in the tail to tail orientation (Fig. 3).

### Effect of Tum1 on the lipid content in *S. cerevisiae*

**Fig. 4:**
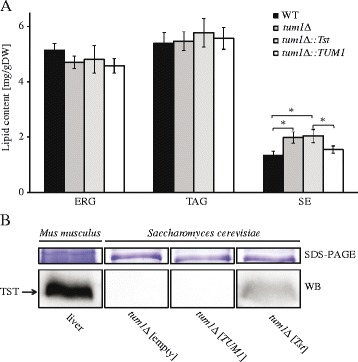
Yeast Tum1 and mouse *Tst* affect lipid metabolism in the *Saccharomyces cerevisiae* cells. **a** Lipid analysis of neutral lipids was performed by TLC. Three different groups of lipids were measured: ergosterol (ERG), triacylglycerols (TAG) and sterol esters (SE). The error bars represent standard deviation. Deletion of *TUM1* causes a 60% increase of SE, as compared to the wild-type. This phenotype is also observed in the strain expressing *Tst*, indicating that *Tst* is not able to complement the loss of *TUM1*. **b** Western blot analysis confirms the presence of *Mus musculus* TST in the cells of *Saccharomyces cerevisiae*. Extracts of proteins were prepared and analysed by polyacrylamide gel electrophoresis and by western blotting with anti-TST antibodies. The *Mus musculus* liver protein sample was used as a positive control and *tum1Δ* and *tum1Δ*::*TUM1* as negative controls. Coomassie Brilliant Blue staining of proteins in the gel with the same amount of protein as for the western blot analysis was used as a loading control. Boxes denote different parts of either the same gel (SDS-PAGE) or membrane (WB)

Next, we investigated the involvement of *TUM1* in lipid metabolism of *S. cerevisiae*. Three different groups of lipids were measured, by TLC: ergosterol (ERG), triacylglycerols (TAG) and sterol esters (SE). The lipid content was first compared in the WT and the *tum1*Δ deletion strains of *S. cerevisiae*. The amount of SE was 60% higher in the deletion than in the WT strain (Fig. 4a). In contrast, the TAG content did not significantly (*p* > 0.05) differ between the cells of these two strains, and ERG levels were also comparable (Fig. 4a).

### Replacement and functional complementation of yeast *TUM1* gene by its mouse homologue *Tst*

To address the question whether the mammalian orthologue can complement the role of *TUM1*, we expressed the mouse *Mus musculus* TST protein in the *tum1*Δ strain, either from a plasmid under the control of a strong promoter (*tum1*Δ[*Tst*] strain) or integrated in the chromosome under the control of the *TUM1* endogenous promoter (*tum1Δ*::*Tst* strain). Western blot analysis was used to confirm the presence of the mouse TST protein in the *tum1*Δ strain transformed with a plasmid containing mouse *Tst* gene, showing the expected band at 33 kDa (Fig. 4b).

Comparison of the lipid content of the *tum1Δ*::*TUM1* and *tum1Δ*::*Tst* strains showed that the SE content of the cells expressing the protein encoded by the mammalian orthologue was significantly (*p* < 0.05) different from that in the strains expressing *TUM1*, but no different than in the *tum1Δ* deletion strain (Fig. 4a). Re-insertion of *TUM1*, on the other hand, resulted in the same lipid content as in the WT strain.

### Effect of Tum1 and Tst on tRNA thiolation status in *S. cerevisiae*

**Fig. 5:**
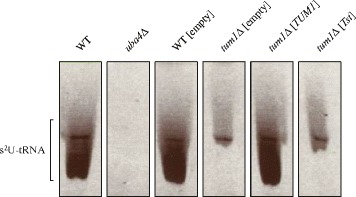
Mouse TST protein cannot complement Tum1 deficiency in the tRNA thiolation pathway. The tRNA thiolation status was analysed in the wild-type strain (WT), the strain without Uba4 protein (*uba4Δ*), and in strains transformed with empty plasmids (WT[empty], *tum1Δ*[empty]) and with plasmids with inserted yeast and mouse homologues (*tum1Δ*[*TUM1*], *tum1Δ*[*Tst*]). Total tRNA was resolved by urea polyacrylamide-gel electrophoresis in the presence of APM

To further investigate functional conservation of *TUM1* and *Tst* genes, we focused on the only hitherto known biological process in which Tum1 is involved, i.e. the pathway for 2-thiolation of the wobble uridine base of the specific tRNA molecules, tE^UUC^, tK^UUU^ and tQ^UUG^. We assessed the tRNA thiolation status in the WT strain, the *uba4*Δ strain (positive control), the WT strain transformed with the empty plasmid (WT[empty]), the *tum1*Δ deletion strain with the empty plasmid (*tum1*Δ[empty]), and in the *tum1*Δ deletion strains transformed with a plasmid containing yeast *TUM1* (*tum1*Δ[*TUM1*]) or mouse *Tst* (*tum1*Δ[*Tst*]) genes. Loss of the slower-migrating modified-tRNA band was partial in the *tum1*Δ[empty] strain and complete in the *uba4*Δ strain (Fig. 5). The level of thiolation of the tRNAs in the *tum1Δ*[*TUM1*] strain was the same as in the WT. On the other hand, the level of thiolation in the *tum1*Δ[*Tst*] strain was indistinguishable from the *tum1Δ*[empty] strain (Fig. 5).

### Cross-talk between the tRNA thiolation pathway and lipid content in *S. cerevisiae*

Finally, we investigated the putative connection between storage lipid content and the tRNA thiolation in *S. cerevisiae*. The measurement of the storage lipid content in the *uba4*Δ strain showed that the SE content did not differ from the WT strain (data not shown). To address this putative cross-talk from a different angle, we inserted into the *tum1*Δ deletion strain the pABY1653 plasmid encoding tE^UUC^, tK^UUU^ and tQ^UUG^ tRNAs, which has been shown to rescue the phenotype caused by the deletion of the genes involved in the tRNA thiolation pathway [10]. The amount of SE, however, did not differ from the *tum1*Δ deletion strain or from the same strain transformed with an empty (control) plasmid. Additionally, we determined that the SE content in the *uba4*Δ deletion strain overexpressing the three tRNAs molecules was the same as in the *uba4*Δ deletion control strain.

## Discussion

The purpose of this study was to determine evolutionary relationships in the rhodanese superfamily and to investigate the molecular function of one superfamily member, Tum1 protein, encoded by the yeast homologue of the mammalian *Tst* gene. To establish evolutionary relationships between *TUM1*, *Tst*, and its paralogue *Mpst*, the analysis was done both at the global level, including genomes from bacteria, archaea and eukaryotes, and at the level of animals and fungi (*Opisthokonta*). The protein sequences in the rhodanese superfamily are challenging for a phylogenetic analysis, as they are divergent and produce low bootstrap values in the phylogenetic trees. We found that the reconstruction of evolutionary relationships in the rhodanese superfamily is relatively simple on the short evolutionary timescale (e.g., in *Opisthokonta*), but it becomes more difficult on the larger evolutionary timescale. In the reconstruction of the evolutionary history in the rhodanese superfamily it was helpful that prokaryotes and eukaryotes contain only a single lineage. The analysis demonstrated that rhodanese superfamily genes are widespread in bacteria, archaea and in all major eukaryotic groups. The prokaryotic sequences do not form a monophyletic clade, but are instead scattered among the eukaryotic representatives, indicating the possibility of horizontal gene transfer, which is further supported by relatively high levels of sequence identity between some prokaryotic and eukaryotic rhodanese representatives.

Tum1 and TST/MPST proteins are representatives of the multidomain sulfurtransferase (rhodanese) superfamily [33]. According to this database, yeast *S. cerevisiae* possesses 11 proteins that belong to the rhodanese superfamily. These proteins belong to three protein families, to the “multidomain sulfurtransferase (rhodanese)” (Tum1, Rdl1, Rdl2 and Ubp7), to the “cell cycle control phosphatase, catalytic domain” (Doa4, Ptp3, Ubp5, Ych1, Mih1 and Arr2) and to the “single domain sulfurtransferase” (Uba4). Tum1 and TST possess the same domain organization of two duplicated TST repeat domains, while the other yeast representatives of the multidomain sulfurtransferase (rhodanese) superfamily – Rdl1, Rdl2 and Ubp7 – are multidomain proteins with the fused rhodanese domain. In contrast to yeast, humans contain a much larger number of proteins that belong to the rhodanese superfamily, i.e. 71 proteins in total according to the Superfamily database [33].

Synteny analyses can be a powerful tool for establishing gene homology relationships and for providing clues about the mechanisms of origins of new genes, as the order and orientation of genes tend to remain the same over relatively long evolutionary time. We therefore reconstructed the ancestral state of the chromosomal positions of the rhodanese superfamily genes. Analysis of conserved syntenies showed clearly that the gene duplication of the rhodanese superfamily occurred ~ 359 My ago in the ancestor of land vertebrates, producing *Tst* and *Mpst* genes.

Having established a direct orthologous relation between *TUM1* and *Tst* genes, we addressed the question of their functional similarity. We found that the amount of SE, but not of TAG or ERG, was significantly higher in the strain lacking *TUM1*. The increased levels of SE in the mutant strain indicate higher activity of the ergosterol pathway, resulting in storage of excess ERG as SE. Namely, the main role of SE is assumed to be storage of excess ERG, in order to keep the concentration of ERG in the membrane constant. According to this interpretation, Tum1 inhibits the ergosterol biosynthetic pathway. Another explanation is also possible, however: while the difference in the ERG content between the WT strain and the strain lacking *TUM1* is not significant, our data indicate that it may be slightly lower in the mutant, meaning that the total ergosterol levels (i.e. ERG + ergosterol in SE) do not differ so considerably between the strains. The difference in distribution of ERG could be a consequence of difference in the cell size, resulting in different membrane surface for the same biomass. Indeed, we observed that cells of the *tum1*Δ strain are approximately 55% (*p* < 0.01) larger than those of the WT strain, in accordance with the latter interpretation. Still, such redistribution of ERG between membranes and as SE in lipid droplets has not been described for other strains which differ in cell size from the WT, and, based on our data, it can only be assumed that Tum1 has a direct effect on the ergosterol pathway. These results thus indicate that Tum1 is indeed involved in lipid metabolism, specifically in the control of the squalene and/or ergosterol pathway.

To determine whether *TUM1* and *Tst* genes remained functionally complementary over the 1.2 billion to 1.5 billion years of evolution [34], we replaced the yeast nonessential *TUM1* gene with the mouse *Tst*, and assayed for the complementation of altered SE content. However, mouse TST could not complement the function of yeast Tum1 protein in its lipid metabolism-related function. Focusing on the pathway for 2-thiolation of the wobble uridine base of the specific tRNA molecules, which hitherto is the only known biological process in which Tum1 is involved, a similar result was obtained. Tum1 is not absolutely required for the biosynthesis of 2-thiouridine, contrasting its role to that of Uba4, but its activity is necessary for wild-type levels of 2-thiouridine [9–11]. On the other hand, mouse TST protein has never been implicated in the tRNA thiolation pathway. The level of tRNA thiolation was only partially decreased in the *tum1*Δ deletion strain, whereas it was completely absent in the *uba4*Δ strain, in agreement with previous findings [9, 10]. Tum1 deficiency in the thiolation pathway was, however, not complemented by the introduction of the mouse homologue *Tst*. We thus demonstrated that the mouse TST protein cannot functionally replace Tum1 deficiency in the tRNA thiolation pathway in yeast, too. It should be noted, however, that even when orthologues perform similar functions in different organisms, it may not be possible to replace one for the other, particularly if the organisms are as widely diverged as mouse and yeast [35]. This result therefore cannot be extended to imply the molecular function of Tst in mammals.

Are the roles of Tum1 in lipid metabolism and in tRNA modification directly linked? We found no evidence of the effect of deletion of *UBA4* on lipid content. Since Uba4 is downstream from Tum1 in the tRNA thiolation pathway [9, 10], this strongly indicates that tRNA thiolation and storage lipid content pathways are not connected. In addition, over-expression of tRNAs that can rescue the phenotype caused by the deletion of the genes involved in the tRNA thiolation pathway, did not rescue or suppress the lipid phenotype of the *tum1*Δ deletion strain, nor did it alter the lipid content in the *uba4*Δ deletion strain. These results strongly support the conclusion that the tRNA thiolation pathway is not involved in the regulation of SE content in *S. cerevisiae*. Notably, the existence of a function for Tum1 in an additional, yet currently unknown pathway has been proposed previously [10].

## Conclusion

The aim of functional genomics has been to determine the function of all the genes comprising a given genome. As several well-studied examples have shown, the property of executing more than one molecular function is far from exceptional. In light of this, determining all the functions for a given gene/protein will most likely be one of the next challenges in functional genomics. The importance of this challenge is further exacerbated when the function of orthologous genes is supported or predicted based on the known function of only one from the pair of the orthologues. Moreover, interactions between cellular pathways or processes are sometimes inferred from common annotated genes/proteins. Such interactions can be mistaken when the gene/protein in question exerts several disparate functions. A case in point is the molecular function of the yeast gene *TUM1*, an orthologue of the mammalian gene *Tst* that was proposed as a candidate obesity-resistance gene [7]: we show here that *TUM1* and *Tst* are direct orthologues, yet the mammalian orthologue cannot replace the yeast gene. We also assigned to yeast Tum1 a new function in lipid metabolism, demonstrated by increased SE content in the gene’s deletion mutant strain, which is most likely exerted through the regulation of the squalene and/or ergosterol pathway. We demonstrate that while the molecular mechanism for this effect is not known, it is not applied through the tRNA modification pathway, the previously described process to which *TUM1* has been annotated. While the evolutionary relationships were clearly established here, the functional relations between yeast *TUM1* and mammalian *Tst* will need to be resolved further.

## Abbreviations

APM: N-acryloylaminophenylmercuric chloride
BIC: Bayesian information criterion
ERG: Ergosterol
ORF: Open reading frame
SE: Sterol esters
TAG: Triacylglycerols
TBS: Tris buffered saline
TLC: Thin-layer chromatography
WT: Wild-type
